# Endotoxin Mass Concentration in Plasma Is Associated With Mortality in a Multicentric Cohort of Peritonitis-Induced Shock

**DOI:** 10.3389/fmed.2021.749405

**Published:** 2021-10-29

**Authors:** Didier Payen, Claire Dupuis, Valérie Deckert, Jean-Paul Pais de Barros, Anne-Laure Rérole, Anne-Claire Lukaszewicz, Remi Coudroy, René Robert, Laurent Lagrost

**Affiliations:** ^1^UFR de Médecine Lariboisière-Saint-Louis, University Paris 7 Denis Diderot, Paris, France; ^2^Medical Intensive Care Unit, Gabriel Montpied University Hospital, Clermont-Ferrand, France; ^3^Inserm, LNC-UMR1231, Dijon, France; ^4^University Bourgogne-Franche Comté, LNC-UMR1231, Dijon, France; ^5^LabEx LipSTIC, FCS Bourgogne-France Comté, Dijon, France; ^6^CHU Dijon, Service de la Recherche, Dijon, France; ^7^Department of Medical Intensive Care, La Miléterie University Hospital, Poitiers University, Poitiers, France

**Keywords:** septic shock, LPS mass, lipoprotein, plasma lipids, phospholipid transfer protein, peritonitis

## Abstract

**Objectives:** To investigate the association of plasma LPS mass with mortality and inflammation in patients with peritonitis-induced septic shock (SS).

**Design:** Longitudinal endotoxin and inflammatory parameters in a multicentric cohort of SS.

**Patients:** Protocolized post-operative parameters of 187 SS patients collected at T1 (12 h max post-surgery) and T4 (24 h after T1).

**Intervention:** *Post-hoc* analysis of ABDOMIX trial.

**Measurements and Results:** Plasma concentration of LPS mass as determined by HPLC-MS/MS analysis of 3-hydroxymyristate, activity of phospholipid transfer protein (PLTP), lipids, lipoproteins, IL-6, and IL-10. Cohort was divided in low (LLPS) and high (HLPS) LPS levels. The predictive value for mortality was tested by multivariate analysis. HLPS and LLPS had similar SAPSII (58 [48.5; 67]) and SOFA (8 [6.5; 9]), but HLPS showed higher death and LPS to PLTP ratio (*p* < 0.01). LPS was stable in HLPS, but it increased in LLPS with a greater decrease in IL-6 (*p* < 0.01). Dead patients had a higher T1 LPS (*p* = 0.02), IL-6 (<0.01), IL-10 (=0.01), and day 3 SOFA score (*p* = 0.01) than survivors. In the group of SAPSII > median, the risk of death in HLPS (38%) was higher than in LLPS (24%; *p* < 0.01). The 28-day death was associated only with SAPSII (OR 1.06 [1.02; 1.09]) and HLPS (OR 2.47 [1; 6.11]) in the multivariate model. In HLPS group, high PLTP was associated with lower plasma levels of IL-6 (*p* = 0.02) and IL-10 (*p* = 0.05).

**Conclusions:** Combination of high LPS mass concentration and high SAPS II is associated with elevated mortality in peritonitis-induced SS patients.

## Introduction

Lipopolysaccharide (LPS) is the principal components of the outer membrane of Gram-negative bacteria, which are considered a major causal factor in the pathophysiology of septic shock (1). Implication of LPS in the pathogenesis of sepsis, especially for Gram-negative bacterial infection has been reported a long time ago (2, 3) in experimental models (4, 5) and in healthy volunteers treated by a small amount of intravenous LPS (6) or in meningococcemia shocked patients (7). However, whether the initial blood level of LPS is related to outcome in septic shock patients has not been addressed. The clinical evidence for a direct link between LPS level and the initiation, progression, and outcome of severe sepsis remains poor, in particular because of limited technology for measuring LPS mass concentration (2, 3). The LPS level has mainly been determined by LPS activity bioassays, which often lead to false negative results in plasma samples (2). Few randomized clinical trials have tested therapies targeting LPS and/or LPS signaling pathways. A recent phase 3 clinical trial targeting the lipid-A moiety of LPS signaling with an anti-TLR4 drug did not improve survival (8). The use of a polymyxin hemoperfusion device (PMX membrane) to remove circulating endotoxins had inconsistent results (9–13). Recent randomized clinical trials (RCTs) testing PMX hemoperfusion failed to reduce mortality in septic shock patients (10–13). In the present study, we quantified LPS in 187 peritonitis-induced septic shock patients enrolled in the ABDOMIX study (11) *via* a direct method using liquid chromatography coupled to tandem mass spectrometry (HPLC-MS/MS) (14). In addition, we explored the main plasma pathway, which neutralizes and detoxifies plasma LPS, by quantifying LDL and HDL as LPS carriers and measuring the activity of plasma phospholipid transfer protein (PLTP), as an LPS-binding and transfer protein (15–19). The first objective was to investigate the relationship between the LPS mass concentration and patient outcome. The second objective was to measure plasma cytokines and to explore the protective role of lipoproteins and PLTP in neutralizing and detoxifying LPS (16, 19, 20).

## Materials and Methods

### Patients

The present cohort came from a registered protocol (Clinicaltrials.gov NCT01222663) approved by the French Ethical Committee (“Comité de Protection des Personnes” Ile de France IV; 2010-A0004039). This multicenter randomized trial tested the potential benefit of using PMX on mortality compared to conventionally treated patients with septic shock secondary to peritonitis (11). The absence of any indication of a reduced death rate related to PMX use allowed the arms of the trial to be grouped in the present work as a unique enrolled, multicentric, and global cohort.

**Figure 1:**
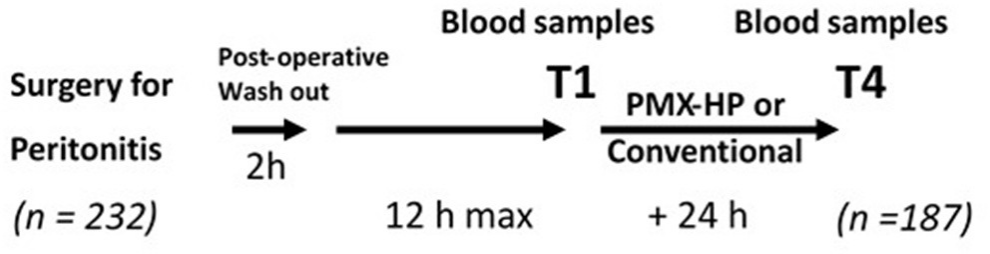
Protocol of the study PMX-HP (Polymyxin-B hemoperfusion). T1, baseline first post-operative measurement; T4, last measurement after PMX-HP or not.

Written informed consent was obtained from patients or their next of kin. The inclusion criteria were: the presence of septic shock using the classic definition (21) related to visually confirmed acute peritonitis (purulent fluid with gut or biliary tract perforation); shock persistence for at least 2 h after anesthesia wash-out, despite treatment by fluid resuscitation (>20 ml/kg) and vasopressor; and an adapted antimicrobial therapy for Gram-negative infection. Demographic characteristics (age, gender), Simplified Acute Physiology Score II at 24 h (SAPS II), the type of surgery, the results from blood or peritoneal culture, and type of antimicrobial therapy were collected. *N* = 232 patients constituted the study group, with a sufficient volume of plasma samples available for laboratory analyses in *N* = 187 patients (Figure 1). Routine laboratory examinations were performed on a day-to-day basis to calculate the Sequential Organ Failure Assessment (SOFA) score. The plasma levels of cholesterol, triglycerides, HDL, and LDL were measured using commercially available kits (Thermo Fisher Scientific, Finland) on an Indiko Clinical Chemistry analyzer (Thermo Fisher Scientific, Finland) according to the manufacturer's instructions. Residual plasma samples were kept frozen at −80°C until more specific measurements could be performed.

### LPS Mass Concentration

The LPS mass concentration was determined by direct quantitation of 3-hydroxymyristic acid (or 3HM) using HPLC-MS/MS as described previously (14). Seven 3HM standards were prepared using a pool of human plasma spiked with 3-hydroxymyristic acid (0, 10, 20, 40, 80, 160, and 320 pmol/ml, respectively). Fifty μl of standards or plasma samples were mixed with 50 μl of saline, 4 pmol of internal standard (IS, 3-hydroxytridecanoic acid 1 pmol/μl in ethanol), and finally hydrolyzed with 300 μl of HCl 8 M for 3 h at 90°C. Total fatty acids were extracted with 600 μl of distilled water and 5 ml of ethyl acetate/hexane (3:2 v/v). After recovery and vacuum evaporation of the organic upper phase, fatty acids were dissolved in 200 μl of ethanol and transferred into 200 μl micro-inserts for LC vials. After evaporation of ethanol under vacuum, samples were finally dissolved in 50 μl of ethanol prior to HPLC/MS/MS analysis (injection volume 2 μl). Fatty acid separation was performed on an Infinity 1260 HPLC binary system (Agilent) equipped with a ZORBAX SB-C18 C18 50 × 2.1 mm 1.8 μm (Agilent) maintained at 45°C with a binary set of eluents (eluent A: ammonium formate 1M/formic acid/water 5/1/996 v/v/v and eluent B: ammonium formate 1M/formic acid/water/acetonitrile 5/1/44/950 v/v/v) at a flow rate of 0.4 ml/min. An 8-min eluent gradient was established as follows: from 0 to 0.5 min, the mobile phase composition was maintained at 45% B; then the proportion of B was increased linearly up to 100% in 2.5 min and maintained for 5 additional minutes. The column was equilibrated with 45% B for 5 min after each sample injection. MS/MS analysis was performed on a 6490 triple quadruple mass spectrometer (Agilent) equipped with a JetStream-Ion funnel ESI source in the negative mode (gas temperature 290°C, gas flow 19 l/min, nebulizer 20 psi, sheath gas temperature 175°C, sheath gas flow 12 l/min, capillary 2,000 V, V charging 2,000 V). Nitrogen was used as the collision gas. The mass spectrometer was set up in the selected reaction monitoring (SRM) mode for the quantification of selected ions as follows: for 3-hydroxytetradecanoic acid, precursor ion 243.2 Da, product ion 59 Da, fragmentor 380 V, collision cell 12 V, cell acceleration 2 V; for 3-hydroxytridecanoic acid, precursor ion 229.2 Da, product ion 59 Da, fragmentor 380 V, collision cell 12 V, cell acceleration 2 V. Areas under the curves of 3HM and IS peaks were determined with Mass Hunter Workstation Software version B.08. The linear correlation method was applied for the calculation of 3HM concentration. LPS mass concentration is expressed as pmol of 3HM per milliliter of plasma.

### Plasma PLTP Activity

PLTP activity was measured using a commercially available fluorescence activity assay from Roar Biomedical (New York, USA) (19, 22). Briefly, plasma (5 μl), fluorescent-labeled donors (3 μl) and unlabeled acceptors (50 μl), were incubated at 37°C in a final volume of 100 μl of assay buffer in 96-well microplates. Changes in fluorescence were monitored using a Victor^3^ multilabel counter (PerkinElmer Life Sciences) with a 465 nm excitation and a 535 nm emission wavelength. This fluorimetric assay measures the transfer (unquenching) of fluorescent phospholipids from donor to acceptor synthetic liposomes. Phospholipid transfer activity was expressed as pmol/h.

### Cytokine Assay

IL-6 (detection limit 1.3 pg/ml) and IL-10 (detection limit 1.6 pg/ml) were measured using the MILLIPLEX MAP Human Cytokine/Chemokine magnetic bead panel kit (Millipore, Darmstadt, Germany) coupled to the Luminex 200 x PONENT software (Luminex, Riverside, CA).

### Statistical Analysis

Comparisons relied on the Fisher exact test for categorical data and the Wilcoxon test for continuous data. *P* < 0.05 was considered significant. The comparisons included patients with a low (LLPS) vs. high (HLPS) level of LPS at baseline, dead or alive at day 28, and low vs. high level (median) of PLTP activity. LLPS and HPLS subgroups were defined on the two sides of the 37.7 pmol/ml 3HM median value.

The risk factors for death at day 28 were investigated by a multivariate logistic regression analysis. The continuous variables were dichotomized and the only variables with a *p* < 0.20 in the univariate analysis were entered into the multivariate model using stepwise selection (*p* < 0.05 considered significant). Missing data were imputed with the median and mode for quantitative and qualitative variables, respectively. All analyses were performed using SAS software, version 9.3 (SAS Institute Inc., Cary, North Carolina).

## Results

Figure 1 shows the study design with the timing for data collection. The baseline characteristics of the whole cohort of 187 patients with available plasma samples are given in Supplementary Table 1. The median SAPSII score was 58 (IQR 48.5; 67), and the median SOFA score excluding the neurological component was 8 (IQR 6.5; 9). The mortality rate was 21.8% at day 28 and 28.2% at day 90 despite adequate antimicrobial therapy in 81.2% of these patients. The high baseline values for the LPS mass concentration were consistent with those previously reported in patients with sepsis (14) and recently in a cohort of intensive care patients including septic shock patients (23). The distribution of patients according to fixed ranges of plasma LPS levels (Supplementary Figure 1A) indicates that a large proportion of patients had a mass concentration >20 pmol/ml. Plasma cholesterol and triglycerides were dramatically low and associated with low levels of HDL and LDL cholesterol and plasma proteins. Conversely, IL-6 and IL-10 were above normal values, indicating an intense systemic inflammatory response (Supplementary Table 1).

Table 1 compares the LLPS and HLPS groups, which were defined using the median LPS value 37.7 pmol/ml. The clinical data were similar between the groups, with a significantly higher rate of mortality in the HLPS group than the LLPS group at day 28 and 90. The plasma PLTP activity level was also higher in the HLPS group than the LLPS group (Table 1), with a significant positive correlation between LPS levels and PLTP values at T1 and T4 (Supplementary Figures 1B,C). Plasma lipid and cytokine concentrations did not differ between these subgroups, except for plasma cholesterol, which was lower in the LLPS group than the HLPS group. The decrease in plasma IL-6 from T1 to T4 was significantly more pronounced in the LLPS group than the HLPS group, despite a significant and concomitant increase in LPS. The LPS to PLTP ratio was lower in the LLPS group than the HLPS group, suggesting more efficient LPS neutralization.

**Table 1:** Comparison between patients with low (lower median value) vs. high (higher median value) of LPS at T1 (baseline) (*n* = 187).

**Variable [median (IQR) or *n* (%)]**	**Low LPS (<37.7) (*n* = 93)**	**High LPS (>37.7) (*n* = 94)**	***P*-value**
PMX-HP	45 (48.4)	49 (52.1)	0.61
Age	72 [63; 78]	73 [64; 79]	0.54
Sex (male)	53 (57)	50 (53.2)	0.60
SAPS II	56 [48; 66]	61 [49; 69]	0.14
SOFA^*^ at day 0	8 [6; 9]	8 [7; 10]	0.12
SOFA^*^ at day 3 (miss = 11)	7 [6; 9]	8 [5; 10]	0.68
**Source of the infection**			
Undetermined	15 (16.1)	11 (11.7)	<0.01
Biliary peritonitis	1 (1.1)	13 (13.8)	
Lower GI perforation	63 (67.7)	43 (45.7)	
Upper GI perforation	14 (15.1)	27 (28.7)	
Nosocomial	56 (60.2)	50 (53.2)	0.33
**Treatments characteristics**			
Adequate antibiotic therapy (miss = 2)	75 (81.5)	76 (81.7)	0.93
**Microbiological findings**			
Gram negative bacteria	64 (68.8)	58 (61.7)	0.31
Gram positive bacteria	44 (47.3)	47 (50)	0.71
Fungi	16 (17.2)	12 (12.8)	0.40
No isolation	8 (8.6)	11 (11.7)	0.48
**Outcomes**			
Mortality at day 28	13 (14)	28 (29.8)	<0.01
Mortality at day 90	16 (17.2)	36 (38.3)	<0.01
**Biomarkers**			
LPS (pmole/ml)	28.1 [22.7; 33]	52.5 [44.3; 70]	<0.01
PLTP T1	431.1 [307.9; 589.4]	487.6 [408; 643.8]	<0.01
Ratio LPS to PLTPT1 (%)	6.2 [4.8; 8.9]	11.5 [8.6; 15.1]	<0.01
Cholesterol T1	0.4 [0.3; 0.6]	0.5 [0.4; 0.7]	0.02
HDL T1	0.1 [0; 0.1]	0.1 [0; 0.2]	1.00
LDL T1	0.1 [0; 0.1]	0.1 [0; 0.1]	0.99
TG T1	0.6 [0.4; 1.1]	0.7 [0.5; 1.2]	0.14
IL10 T1 (miss = 8)	205.9 [69.5; 865.5]	211.5 [87.2; 557.6]	0.59
IL6 T1 (miss = 8)	2102.3 [557.2; 9212.1]	1583.2 [388.2; 8264.9]	0.35
Delta (T4–T1) LPS (miss = 22)	14.1 [4.6; 23.7]	1.5 [−9.8; 26]	<0.01
Delta (T4–T1) IL10 (miss = 24)	−75.2 [−327.9; −10.9]	−68 [−266.8; −0.9]	0.21
Delta (T4–T1) IL6 (miss = 24)	−1414.7 [−4332.7; −358.4]	−509.4 [−3681.1; −72.7]	0.01

*IQR, Interquartile range; SAPS, simplified acute physiologic score; PMX-HP, Polymyxin-B hemoperfusion; T1, baseline; PLTP, Phospholipid transfer protein; IL, Interleukin; Delta, variations from baseline (T1) to (T4) (T4-T1)*.

* *SOFA score excluding the neurological alteration*.

Table 2 compares dead and alive patients at day 28. The patients who died had higher baseline LPS and a greater increase in LPS levels over time compared to surviving patients. The baseline LPS to PLTP ratio and inflammatory cytokines were also higher in patients who died.

**Table 2:** Comparison between the groups of patients alive or dead at day 28 (*n* = 187).

**Variable (median [IQR) or *n* (%)]**	**Alive at day 28 (*n* = 147)**	**Dead at day 28 (*n* = 41)**	***P*-value**
PMX-HP	71 (48.3)	23 (56.1)	0.38
Age	71 [61; 78]	77 [67; 79]	<0.01
Sex (male)	83 (56.5)	21 (51.2)	0.55
SAPS II	56 [48; 64]	67 [61; 79]	<0.01
SOFA^*^ at day 0	8 [6; 9]	9 [7; 10]	0.08
SOFA^*^ at day 3 (miss = 11)	7 [5; 9]	9 [7; 10]	0.01
**Source of the infection**			
Undetermined	21 (14.3)	5 (12.2)	0.26
Biliary peritonitis	8 (5.4)	6 (14.6)	
Lower GI perforation	86 (58.5)	21 (51.2)	
Upper GI perforation	32 (21.8)	9 (22)	
Nosocomial	81 (55.1)	26 (63.4)	0.34
**Treatments characteristics**			
Adequate antibiotic therapy (miss = 2)	116 (80)	35 (85.4)	0.46
**Microbiological findings**			
Gram negative bacteria	93 (63.3)	29 (70.7)	0.38
Gram positive bacteria	68 (46.3)	23 (56.1)	0.26
Fungi	21 (14.3)	7 (17.1)	0.66
No isolation	16 (10.9)	3 (7.3)	0.50
**Biomarkers**			
LPS (pmole/ml) T1 (miss = 1)	36.5 [27; 48.1]	45.5 [35.1; 63.1]	0.02
PLTP T1 (miss = 1)	469.8 [366.7; 587.9]	437.8 [327.5; 715.9]	0.88
Ratio LPS to PLTP T1 (%) (miss = 1)	8.4 [5.8; 11.5]	10.1 [7; 14]	0.03
Cholesterol T1 (miss = 1)	0.5 [0.3; 0.6]	0.4 [0.3; 0.6]	0.18
HDL T1 (miss = 1)	0.1 [0; 0.2]	0.1 [0; 0.1]	0.91
LDL T1 (miss = 1)	0.1 [0; 0.1]	0.1 [0; 0.1]	0.40
TG T1 (miss = 1)	0.7 [0.5; 1.2]	0.7 [0.5; 0.9]	0.58
Protein T1 (miss = 1)	33.9 [29.6; 38.8]	32.6 [27.2; 36.3]	0.10
IL-10 T1 (miss = 9)	170 [69.6; 453.9]	432.5 [134.2; 1,247.6]	0.01
IL-6 T1 (miss = 9)	1524.9 [474.5; 7796.8]	7134.4 [1,083.6; 14190.1]	<0.01
Delta (T4-T1) LPS (miss = 23)	7.8 [−2.7; 21.2]	24.6 [−5.5; 51]	0.03
Delta (T4-T1) PLTP (miss = 24)	151.8 [58.1; 325]	117.2 [31.5; 258.1]	0.28
Delta (T4-T1) IL10 (miss = 25)	−82.3 [−319.5; −11.5]	−19.6 [−168.5; 90.3]	0.02
Delta (T4-T1) IL6 (miss = 25)	−862.8 [−4038.7; −235.9]	−840.7 [−5458.9; 0]	0.51

*IQR, Interquartile range; SAPS, simplified acute physiologic score; PMX-HP, Polymyxin-B hemoperfusion; T1, baseline; PLTP, Phospholipid transfer protein; IL, Interleukin; Delta, variations from baseline (T1) to (T4) (T4-T1)*.

* *SOFA score excluding the neurological alteration*.

**Figure 2:**
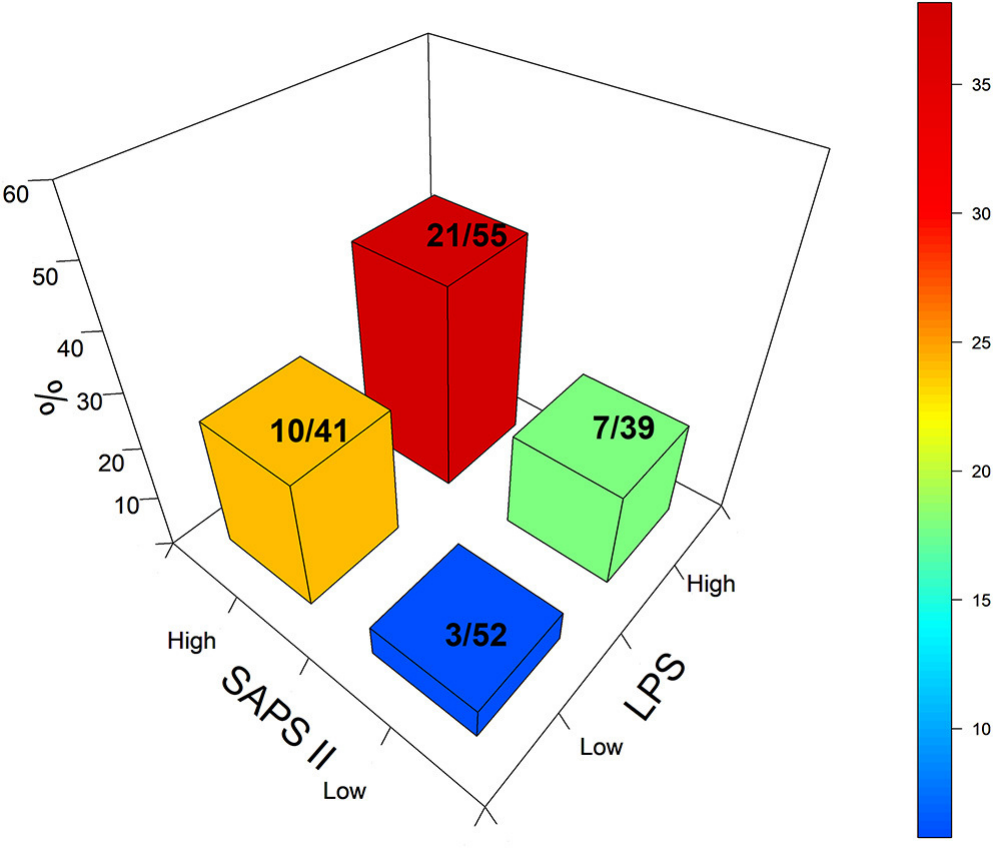
Tri-dimensional representation of death rate based on high and low SAPS II scores 24 h after admission and the baseline LPS level. The numbers correspond to the absolute deaths over the total number for each category. Blue: Low SAPS II, low LPS: 5.7% of deaths. Green: High LPS, low SAPS II: 17.9% of deaths. Orange: Low LPS, high SAPS II: 24% of deaths. Red: high LPS; high SAPS II: 38.1% of deaths.

Univariate analysis showed that age, SAPSII, and high levels of LPS and IL-6 at T1 were associated with mortality. The stepwise multivariate analysis (Supplementary Table 2) validated only SAPS II and high or low LPS level as independent predictors of mortality at day 28. The Figure 2 illustrates the impact of all combinations between SAPS II and LPS mass values partitioned in low and high values referring to the median LPS value observed in the whole cohort. Death rate with high SAPS II or high LPS alone (17.9 and 24.2%, respectively) were significantly lower than the rate of death when high SAPS II and LPS were combined (38%; *p* < 0.01).

Table 3 compares the patients with low vs. high T1 PLTP activity. With the exception of sex distribution (*p* < 0.05), the clinical characteristics of the groups were similar, particularly the mortality rate at day 28 and 90. However, despite the higher T1 LPS level in the high PTLP group vs. the low PLTP group, the LPS to PLTP ratio was lower with a lower IL-6 plasma level.

**Table 3:** Comparison between the patients having a low (infra median) vs. high (supra median) level of PLTP on the whole cohort (*n* = 187).

**Variable**	**Low PLTP (*n* = 93)**	**High PLTP (*n* = 94)**	***P*-value**
PMX-HP	45 (48.4)	49 (52.1)	0.61
Age	71 [64; 78]	73 [63; 79]	0.26
Sex, male	58 (62.4)	45 (47.9)	0.05
SAPS II	57 [48; 67]	58 [50; 67]	0.60
SOFA^a^ at day 0	8 [6; 9]	8 [7; 10]	0.24
SOFA^a^ at day 3 (missing = 11)	7 [6; 9]	7 [5; 9]	0.72
**Source of the infection**			
Undetermined	13 (14)	13 (13.8)	0.09
Biliary peritonitis	4 (4.3)	10 (10.6)	
Lower GI perforation	60 (64.5)	46 (48.9)	
Upper GI perforation	16 (17.2)	25 (26.6)	
Nosocomial	51 (54.8)	55 (58.5)	0.61
**Treatment characteristics**			
Adequate antibiotic therapy (missing = 2)	74 (81.3)	77 (81.9)	0.54
**Microbiological findings**			
Gram-negative bacteria	58 (62.4)	64 (68.1)	0.41
Gram-positive bacteria	44 (47.3)	47 (50)	0.71
Fungi	13 (14)	15 (16)	0.70
No isolation	12 (12.9)	7 (7.4)	0.22
**Outcomes**			
Mortality at day 28	23 (24.7)	18 (19.1)	0.36
Mortality at day 90	27 (29)	25 (26.6)	0.71
**Biomarkers**			
LPS T1, pmole/ml	36.1 [26.2; 46.9]	43.6 [29.5; 56.4]	0.01
PLTP T1	357.9 [283.3; 414.2]	600.8 [520.1; 731.7]	<0.01
LPS:PLTP ratio T1, %	10.5 [8.2; 14]	6.8 [4.9; 9.6]	<0.01
Cholesterol T1	0.4 [0.3; 0.5]	0.6 [0.4; 0.7]	<0.01
HDL T1	0.1 [0; 0.2]	0.1 [0; 0.1]	0.87
LDL T1	0.1 [0; 0.1]	0.1 [0; 0.1]	0.86
TG T1	0.6 [0.4; 0.9]	0.8 [0.5; 1.2]	<0.01
Proteins T1	32.5 [26; 37.8]	34.3 [30.5; 38.8]	0.04
IL-10 T1 (missing = 8)	263.3 [77.2; 979.5]	179.1 [76.4; 414.8]	0.16
IL-6 T1 (missing = 8)	3807.1 [692.7; 10,315.9]	1195 [429; 6504.1]	<0.01
Delta LPS (missing = 22)	10 [1; 24.6]	9.9 [−4.8; 21.5]	0.42
Delta PLTP (missing = 23)	208.5 [101.6; 362.6]	91.5 [−13.4; 232.4]	<0.01
Delta LPS/PLTP ratio, % (missing = 24)	−2.5 [−4.4; 1]	0.2 [−1.7; 2.5]	<0.01
Delta IL-10 (missing = 24)	−82.3 [−413.1; −10.5]	−69.1 [−270.1; −1.8]	0.34
Delta IL-6 (missing = 24)	−1409.5 [−5951.2; −293.8]	−733 [−3653.8; −123.2]	0.06

*Data are presented as median (interquartile range) or n (%)*.

*SAPS, Simplified Acute Physiological Score; PMX-HP, polymyxin-B hemoperfusion; T1, baseline; PLTP, phospholipid transfer protein; IL, interleukin; Delta, variations from baseline (T1) to (T4) (T4-T1)*.

a *SOFA score excluding the neurological alteration*.

Supplementary Table 3 depicts the impact of PLTP in the high T1 LPS mass. Although the mortality rates were not different, the high PLTP group had a lower LPS to PLTP ratio and lower plasma levels for both IL-6 and IL-10.

## Discussion

As a primary end point, the detection of high levels of LPS mass determined by a direct HPLC-MS/MS assay at a similar SAPS II is associated with higher mortality. As a secondary end point, the elevation of plasma PLTP levels was found associated with a reduced systemic inflammation assessed by IL-6 and IL-10 plasma levels.

With regard to the primary results of this study, the patients with a supra-median level of LPS (HLPS group) at baseline had a higher rate of death at day 28 and 90 compared to the patients with an infra-median level (LLPS group), despite similar clinical characteristics at admission. These results extend a previous study from our group in a mixed cohort of ICU patients with SIRS, no SIRS, sepsis or septic shock from different origins (23). For the first time in human septic shock related to peritonitis, we report here that a high level of LPS is an additional risk factor for death in patients with the highest SAPS II score, i.e., with high severity. Notably, we did not find a relationship between the baseline LPS mass concentration and the presence of Gram-negative bacteria in culture (64.9%), with a relatively high incidence of Gram-positive bacteria. This apparent contradiction may result from the selected septic phenotype. Septic shock was frequently shown to be associated with an abnormal high capillary permeability, a condition that favors translocations of bacteria similar or not to the initial one (24). A large proportion of patients had LPS levels close to the 37.7 pmole/ml median value, whereas some patients had very high LPS levels (Figure 2). The HLPS and LLPS subgroup comparison confirmed the relationship between the baseline LPS mass concentration, the severity of shock, and mortality. The combination of high SAPSII score and high LPS mass concentration corresponded to the worse prognosis. Despite similar clinical data (SAPSII and SOFA scores) and distributions of Gram-negative and Gram-positive infections, the mortality rate was remarkably different between the high and low LPS groups at both day 28 and 90. This study comes in support of a relationship between the plasma mass concentration of LPS and outcome in septic shock patients. This major link is reinforced by the results of the multivariate analysis, showing a high OR for both LPS > 37.7 pmole/ml and SAPS II score being associated with a worse outcome. The kinetics of LPS over 48 h were also different in the two subgroups. If the high LPS level was stable in the supra-median subgroup, it gradually increased in the infra-median subgroup, but it did not translate into a worsening of the inflammatory trait. Despite similar baseline plasma IL-6 and IL-10 levels within the two groups, the faster decrease in plasma IL-6 in the LLPS group compared to the HLPS group supports the benefit to have a low-to-moderate LPS level. The covariable PMX never differed, including for the blood LPS mass concentration, which fits well with the negative results of the ABDOMIX trial and the ancillary study looking at cytokine levels as previously reported (11, 25).

With regard to the secondary results of this study, a higher LPS:PLTP ratio (used as an index of poor capability to neutralize LPS) was associated with higher inflammation in non-survivors compared to survivors. In the meantime, no significant differences in plasma lipid and lipoprotein parameters were observed between survivors and non-survivors. It is consistent with previous mouse studies which reported that the binding capability of plasma PLTP, not the lipoprotein pool size, is critical to repress LPS-induced inflammation (22). In addition, a high PLTP level was associated in the present study with lower IL-6 levels, despite elevated LPS levels. Again, it is in line with the ability of PLTP, as a member of the lipid transfer/lipopolysaccharide binding protein family, to bind and transfer LPS (15, 16, 18, 19, 26). It also agrees with *in vitro* and *in vivo* studies in preclinical models [including PLTP-knocked out mice and the infusion of active recombinant human PLTP in mice with experimental sepsis (15, 19, 22, 27)] which have revealed that the PLTP-mediated transfer of LPS to lipoproteins results in neutralization of the pro-inflammatory properties of LPS and in its elimination from the body. Elevated PLTP activity was also reported in patients with severe sepsis (28, 29) and during the acute-phase response induced by infection (26). Taken together, the previous reports and the present results strongly suggest that PLTP activity may adapt to and eliminate the culprit endotoxins in patients with septic shock. One can then reasonably hypothesize that early detection of elevated LPS mass concentration relates to severe inflammation and poor prognosis, especially if PLTP activity is insufficient. This hypothesis is in agreement with the worse outcome observed when elevated LPS mass concentration and high SAPS II are present together. Further studies are needed for more direct evidence.

Our study has some limits and advantages. As a limitation, and at this stage, only a few studies from one single group were conducted using the direct quantitation of 3HM by HPLC-MS/MS analysis, and mean 3HM concentration values varied among distinct reports [(14, 23), present study]. Due to the paucity of information, additional studies from independent laboratories are necessary to reach reference values in distinct populations in which elevated endotoxemia is expected. In addition, the relatively low median value for LPS in the present study may result in part from ample hemodilution, as the low plasma protein level (~32–34 g/L) suggests. This dilution could be consecutive to the pre-operative resuscitation and peri-operative fluid administration, which was not quantified in the present study. Finally, and because 3HM is the most abundant but not unique 3OH-fatty acid found in the lipid A moiety of LPS molecules, the measurement and sum of all 3OH-FAs ranging from 10 to 18 carbons atoms might provide a better assessment of LPS concentration in future studies.

Apart of the limits, the present study has some advantages. First, it is a multicentric study enrolling a homogenous group of septic shock patients. Second, the resuscitation protocol was pre-defined to limit the inter center therapy heterogeneity. Third, the surgical procedure was blindly evaluated by an independent surgeon to validate the surgical procedure. Fourth, the HPLC-MS/MS method used for LPS mass measurement is the only one providing the direct quantitation of LPS in plasma. Fifth, the delay from the onset of sepsis is frequently clear in peritonitis, limiting the heterogenous delay for measurements.

## Conclusion

In the peritonitis-induced septic shock, the elevated LPS mass is associated with higher mortality than SAPS II with low LPS mass. In addition, an elevated PLTP plasma level in high LPS mass may reduce the systemic inflammation as IL-6 plasma levels. It supports the protective role of PLTP in neutralizing LPS in the context of septic shock, and it is in line with previous studies in animal models of endotoxemia or sepsis (15, 19, 27). More clinical studies are warranted to validate the present results and to support the potential use of PLTP molecule as a protecting drug in septic shock.

## Data Availability Statement

The raw data supporting the conclusions of this article will be made available by the authors, without undue reservation.

## Ethics Statement

The studies involving human participants were reviewed and approved by Comité de Protection des Personnes Ile de France IV; 2010-A0004039. The patients/participants provided their written informed consent to participate in this study.

## ABDOMIX Group

- J. Guilhot, Inserm Unit CIC 1402, La Miléterie University Hospital, University Poitiers, Poitiers, France
- Y. Launey, Department of Anesthesia and Critical Care, Pontchaillou University Hospital, University Rennes, Rennes, France
- M. Kaaki, Department of Intensive Care, Hospital of Roanne, Roanne, France
- B. Veber, Department of Anesthesia and Critical Care, University Charles Nicolle Hospital, University Rouen, Rouen, France
- J. Pottecher, Department of Anesthesia and Critical Care, University Hospital Civil of Strasbourg, University Strasbourg, Strasbourg, France
- O. Joannes-Boyau, Department of Anesthesia and Critical Care, Haut-Lévêque University Hospital, University Bordeaux, Bordeaux, France
- L. Martin-Lefevre, Medical-Surgical Intensive Care, District, Hospital, La Roche Sur-Yon, France
- M. Jabaudon, Department of Anesthesia and Critical Care, D'Estaing University Hospital, University Clermont-Ferrand, Clermont-Ferrand, France
- O. Mimoz, Department of Anesthesia and Surgical, Intensive Care, La Miléterie University Hospital, Poitiers University, Poitiers, France
- M. Ferrandière, Department of Anesthesia and Critical Care, Trousseau University Hospital, University Tours, Tours, France
- E. Kipnis, Department of Anesthesia and Critical Care, Huriez University Hospital, University Lille, Lille, France
- C. Vela, Department of Anesthesia and Critical Care, University Hospital, Lille, France
- S. Chevallier, Department of Intensive Care, Hôpital Saint-Jean, Perpignan, France
- J. Mallat, Medical-Surgical Intensive Care, District Hospital, Saint-Malo, France.

## Author Contributions

DP and RR designed the study. LL, VD, J-PP, and A-LR performed the LPS mass and PLTP measurements. DP, RR, RC, and A-CL collected the clinical data and measured the levels. CD did all the statistical study. DP and LL conducted data analysis and wrote the manuscript. VD, RR, and A-CL revised critically the manuscript. All authors agree to be accountable for all aspects of the work.

## Funding

This study was initially sponsored by Torey Medical Ltd by an unlimited grant and had no role in the protocol design, conduct of the study, collection, management, analysis, and interpretation of the data, preparation, review, or approval of the manuscript, and decision to submit the manuscript for publication. The laboratory investigations were supported by the INSERM (Institut National de la Santé et de la Recherche Médicale), the Regional Council of Bourgogne, the European Regional Development Fund, and a French Government grant managed by the French National Research Agency (ANR) under the program Investissements d'Avenir with reference ANR-11-LABX-0021-01-LipSTIC LabEx.

## Conflict of Interest

The authors declare that the research was conducted in the absence of any commercial or financial relationships that could be construed as a potential conflict of interest.

## Publisher's Note

All claims expressed in this article are solely those of the authors and do not necessarily represent those of their affiliated organizations, or those of the publisher, the editors and the reviewers. Any product that may be evaluated in this article, or claim that may be made by its manufacturer, is not guaranteed or endorsed by the publisher.
