# Supplementary material for: Endotoxin Mass Concentration in Plasma Is Associated With Mortality in a Multicentric Cohort of Peritonitis-Induced Shock
Source: Front Med (Lausanne). 2021 Oct 29;8:749405. doi: 10.3389/fmed.2021.749405 (PMC8586519; doi:10.3389/fmed.2021.749405)
Supplement: Supplementary file 1 [file Table_1.DOCX]

Supplementary Table 1: Baseline characteristics of whole cohort of patients

| **Variable (Median (IQR) or n (%))** | **All (N=187)** |
| --- | --- |
| PMX-HP | 94 (50) |
| Age | 72 [63 ; 79] |
| Sex(male) | 104 (55.3) |
| SAPS II | 58 [48.5 ; 67] |
| SOFA* at day 0 | 8 [6.5 ; 9] |
| SOFA* at day 3(miss=11) | 7 [6 ; 9] |
| *Source of the infection* |  |
| Undetermined | 26 (13.8) |
| Biliary peritonitis | 14 (7.4) |
| Lower gastrointestinal tract perforation | 107 (56.9) |
| Upper gastrointestinal tract perforation | 41 (21.8) |
| Nosocomial | 107 (56.9) |
| *Treatments characteristics* | |
| Adequate antibiotic therapy(miss=2) | 151 (81.2) |
| *Microbiological findings* | |
| Gram negative bacteria | 122 (64.9) |
| Gram positive bacteria | 91 (48.4) |
| Fungi | 28 (14.9) |
| No isolation | 19 (10.1) |
| *Outcomes* |  |
| Mortality at day 28 | 41 (21.8) |
| Mortality at day 90 | 53 (28.2) |
| *Biomarkers* |  |
| LPS (pmol/ml) (miss=1) | 37.7 [28.2 ; 52.8] |
| PLTP activity T1 (pmol/h)(miss=1) | 465.3 [361.7 ; 601.8] |
| Ratio LPS to PLTP T1 (%)(miss=1) | 8.85 [6.01 ; 11.84] |
| Cholesterol (g/L) T1(miss=1) | 0.48 [0.343 ; 0.632] |
| HDL (g/L) T1(miss=1) | 0.066 [0.038 ; 0.138] |
| LDL (g/L) T1(miss=1) | 0.058 [0.017 ; 0.118] |
| TG (g/L) T1(miss=1) | 0.677 [0.461 ; 1.105] |
| Protein T1(miss=1) g/L | 33.3 [29.0 ; 38.5] |
| IL-10 T1(miss=9) (pg/ml) | 206.9 [77.2 ; 651.2] |
| IL-6 T1(miss=9) (pg/ml) | 1957.4 [479.0 ; 9212.1] |

* SOFA score excluding the neurological alteration; PMX-HP= Polymyxin-B hemoperfusion; LPS: lipopolysaccharide; T1: baseline time; T4: time corresponding to the end of 2^nd^ hemoperfusion session. IL: Interleukin; IQR= Interquartile range; SAPS: simplified acute physiologic score; PLTP: Phospholipid transfer protein
