# Supplementary material for: Endotoxin Mass Concentration in Plasma Is Associated With Mortality in a Multicentric Cohort of Peritonitis-Induced Shock
Source: Front Med (Lausanne). 2021 Oct 29;8:749405. doi: 10.3389/fmed.2021.749405 (PMC8586519; doi:10.3389/fmed.2021.749405)
Supplement: Supplementary file 2 [file Table_2.DOCX]

Supplementary Table 2: Risk of death at day 28: multivariate analysis

| **Variable** | **OR** | **CI** | **Pvalue** |
| --- | --- | --- | --- |
| Total LPS mass > 37 | 2.76 | [1.23 ; 6.18] | 0.014 |
| SAPS II | 1.07 | [1.04 ; 1.1] | 0.000 |

Among the factors associated with D28 mortality after univariate analysis, only the baseline LPS mass level and SAPS II were significant after multivariate analysis. LPS: lipopolysaccharides; SAPSII simplified acute physiological score; OR: odds ratio; CI: confidence interval
