# Supplementary material for: Endotoxin Mass Concentration in Plasma Is Associated With Mortality in a Multicentric Cohort of Peritonitis-Induced Shock
Source: Front Med (Lausanne). 2021 Oct 29;8:749405. doi: 10.3389/fmed.2021.749405 (PMC8586519; doi:10.3389/fmed.2021.749405)
Supplement: Supplementary file 3 [file Table_3.DOCX]

Supplementary Table 3: comparison of baseline (T1) low (infra median) *vs* high (supra median) level of PLTP among patients with high T1 level LPS mass.

| **Variable (Median (IQR) or (%))** | **PLTP Low (n=41)** | **PLTP high (n=53)** | **P value** |
| --- | --- | --- | --- |
| PMX-HP | 21 (51.2) | 28 (52.8) | 0.88 |
| Age | 68 [61 ; 77] | 75 [68 ; 81] | 0.02 |
| Sex (male) | 25 (61) | 25 (47.2) | 0.18 |
| *Outcomes* |  |  |  |
| Mortality at day 28 | 13 (31.7) | 15 (28.3) | 0.72 |
| Mortality at day 90 | 16 (39) | 20 (37.7) | 0.90 |
| *Biomarkers* |  |  |  |
| LPS (pmole/ml) T1 | 47.9 [41 ; 61.7] | 55 [45.7 ; 72.5] | 0.03 |
| PLTP T1 | 385.8 [349.4 ; 428.6] | 601.8 [513.8 ; 736.9] | <0.01 |
| Ratio LPS to PLTP T1 (%) | 14 [11.6 ; 16.9] | 9.1 [7.6 ; 11.7] | <0.01 |
| IL-10 T1 (miss=4) | 291 [88.2 ; 797.5] | 158.7 [74.2 ; 397.1] | 0.05 |
| IL-6 T1 (miss=4) | 6114.1 [570.3 ; 13804.5] | 864.2 [331.2 ; 4958.7] | 0.02 |
|  |  |  |  |

Data are presented as median (interquartile range IQR) or n (%). IQR= Interquartile range; T1: baseline; LPS: lipopolysaccharides; PTLP Phospholipid transfer protein; IL: Interleukin;

In presence of high basal level of LPS mass, a high basal PLTP reduced the LPS to PLTP ratio suggesting a stronger LPS neutralization. This effect was associated with a reduction in IL-10 and IL-6.
