# Supplementary figures and images for: Endotoxin Mass Concentration in Plasma Is Associated With Mortality in a Multicentric Cohort of Peritonitis-Induced Shock
Source: Front Med (Lausanne). 2021 Oct 29;8:749405. doi: 10.3389/fmed.2021.749405 (PMC8586519; doi:10.3389/fmed.2021.749405)

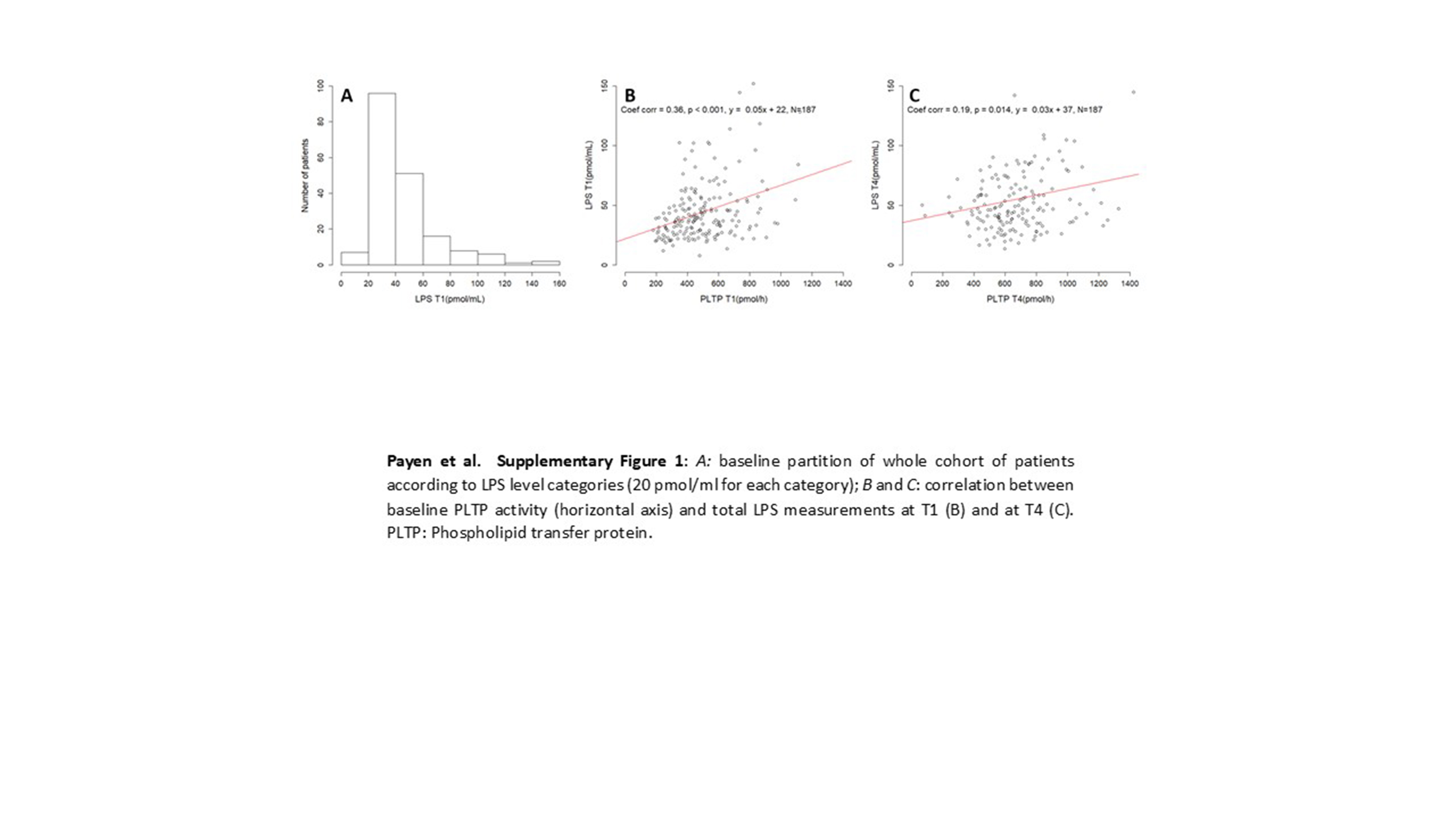

Supplement: Supplementary file 4 [file Image_1.JPEG]
